# Effectiveness of Adding Thoracic Manual Therapy to Conventional Pulmonary Rehabilitation for Improving Pulmonary Function and Chest Expansion in Adults with Restrictive Ventilatory Impairment: A Randomized Controlled Trial

**DOI:** 10.3390/life16091571

**Published:** 2026-09-20

**Authors:** Mshari Alghadier, Hesham A. Alfeheid, Hany M. Elgohary, Ibrahim Ismail Abuzaid

**Affiliations:** 1 Department of Health and Rehabilitation Sciences, Prince Sattam bin Abdulaziz University, Alkharj 11942, Saudi Arabia; h.alfeheid@psau.edu.sa; 2 Department of Physical Therapy, Faculty of Applied Medical Sciences, Jerash University, Jerash P.O. Box 311, Jordan; hmielgohary@gmail.com (H.M.E.); Ibrahim_ismail_2011@svu.edu.eg (I.I.A.); 3 Department of Physical Therapy for Surgery, Faculty of Physical Therapy, Cairo University, Giza 12613, Egypt; 4 Department of Physical Therapy for Cardiovascular/Respiratory and Geriatrics, Faculty of Physical Therapy, Qena University, Qena 83523, Egypt

**Keywords:** pulmonary function, restrictive lung diseases, respiratory muscle training, thoracic mobility, myofascial release techniques

## Abstract

**Background and Objective**: Restrictive ventilatory impairment is characterized by reduced lung volumes and may be accompanied by reduced thoracic mobility. The purpose of this study was to determine if the addition of thoracic manual therapy to conventional pulmonary rehabilitation (PR) was superior to conventional PR alone in improving pulmonary function and chest expansion in adults with restrictive ventilatory impairment. **Methods**: This was a randomized controlled trial (RCT) involving 48 patients with restrictive ventilatory impairment, who were randomly assigned to two groups: conventional pulmonary rehabilitation (Group A, n = 24) and conventional pulmonary rehabilitation with thoracic manual therapy (Group B, n = 24). Forced vital capacity (FVC), as a percentage of the predicted value, was the prespecified primary outcome. Secondary outcomes were forced expiratory volume in 1 s (FEV_1_), total lung capacity (TLC), FEV_1_/FVC ratio, and chest expansion at the 2nd and 4th intercostal spaces and the xiphoid level. Assessment of outcomes was conducted at baseline, after 8 weeks of treatment, and at a 4-week follow-up. **Results**: There were significant group vs. time interactions for FVC, FEV_1_, TLC, FEV_1_/FVC, and chest expansion at each of the thoracic levels measured (all *p* < 0.001), suggesting that the change over time was different among groups. Between-group differences were greatest after intervention and at 4-week follow-up, with Group B showing greater gains than Group A. **Conclusions**: In adults with restrictive ventilatory impairment, thoracic manual therapy in addition to conventional pulmonary rehabilitation resulted in greater improvement in selected pulmonary function and chest-expansion measures at 8 weeks and follow-up than conventional pulmonary rehabilitation alone. These differences were maintained during the four-week follow-up period.

## 1. Introduction

Restrictive lung diseases (RLDs) are a heterogeneous group of respiratory diseases characterized by decreased lung volumes, decreased chest wall compliance, and impaired pulmonary function, and are frequently associated with dyspnea, exercise intolerance, and diminished quality of life [1,2]. In contrast to obstructive lung diseases, where the airflow is mainly impaired, RLDs are characterized by a maintained or even increased FEV_1_/FVC ratio, and a reduced total lung capacity (TLC), indicating a restriction in lung expansion but not airway blockage [3]. Some of the common causes are interstitial lung disease, pulmonary fibrosis, post-surgical changes, scoliosis, obesity, and neuromuscular disorders [4]. The resultant restrictive pattern tends to be related to decreased ventilation of the alveoli, impaired gas exchange, and augmented work of breathing, which, together, leads to functional restrictions and enhanced vulnerability to respiratory complications [5].

A reduced FVC with a preserved or increased FEV_1_/FVC ratio is suggestive of a restrictive spirometric pattern but does not, by itself, establish the presence of true restriction. Confirmation of restrictive ventilatory impairment generally requires demonstration of a reduced total lung capacity [6]. Therefore, in this study, spirometric findings were interpreted together with TLC measurements obtained by whole-body plethysmography. The study population included participants with different clinical conditions capable of producing restrictive ventilatory impairment.

The key factors in the functional impairment of RLDs are respiratory muscle weakness and decreased chest wall compliance [7]. Dysfunction of the diaphragm, stiff intercostals, and lack of mobility of the rib cage restrict effective inspiration and expiration, which decreases vital capacity and tidal volume [8]. Moreover, the primary respiratory muscle insufficiency is frequently amended by other muscles, such as the scalene, sternocleidomastoid, or pectoralis minor, which results in muscular exhaustion and pain [9]. These neurological and musculoskeletal disabilities affect the efficiency of ventilation, limit the exercise capabilities, and decrease the functional autonomy of the affected individuals [10].

Pulmonary rehabilitation has become one of the pillars of treatment in restrictive lung disorders, aiming to optimize the performance of respiratory muscles, increase lung volumes, and ease the clearance of the airways [11]. Different exercises, including diaphragmatic breathing, segmental breathing, active cycle of breathing techniques (ACBT), and incentive spirometry, have shown improvements in FVC, FEV_1_, and chest expansion in restrictive and mixed respiratory pattern patients [12,13]. Moreover, thoracic joint mobilization and myofascial release, as part of the manual therapy intervention, may increase thoracic compliance, ease diaphragmatic excursion, and improve accessory muscle coordination [14,15,16,17]. There is evidence that these interventions can have a beneficial effect on pulmonary function by increasing the mobility of the rib cage, enhancing the biomechanics of the chest wall, and decreasing musculoskeletal limitations that restrict ventilation [18,19].

Despite the potential contribution of thoracic manual therapy to thoracic mobility, evidence regarding its additional value when incorporated into conventional pulmonary rehabilitation in adults with restrictive ventilatory impairment remains limited. Previous research has predominantly investigated manual therapy or thoracic mobilization in populations with obstructive respiratory disease, postoperative conditions, or other specific clinical populations, whereas evidence in heterogeneous non-obstructive restrictive ventilatory impairment is comparatively limited [20]. In particular, few randomized controlled trials have directly compared conventional pulmonary rehabilitation with the same rehabilitation program supplemented by a structured combination of thoracic mobilization and myofascial release while simultaneously assessing pulmonary function and thoracic mobility [21].

Therefore, this trial aimed to determine whether adding thoracic manual therapy, consisting of thoracic mobilization and myofascial release, to conventional pulmonary rehabilitation produces greater improvements in pulmonary function and chest expansion than conventional pulmonary rehabilitation alone. The prespecified primary outcome was FVC (% predicted), while FEV_1_, TLC, FEV_1_/FVC, and chest expansion were evaluated as secondary outcomes. Realizing the cumulative impact of manual therapy may serve as a clinical practice to optimize rehabilitation methods to achieve the best possible lung capacity, chest expansion, and efficient functioning of the respiratory system.

## 2. Materials and Methods

The study was carried out in the outpatient clinic of the Faculty of Physical Therapy, South Valley University, and the participants were recruited from South Valley University Hospitals from February 2025 to March 2025. The study was conducted in accordance with the CONSORT requirements for clinical trials and registered in clinicalTrials.gov (NCT06681701, date: 15 March 2025), with the approval of the Institutional Research Ethics Committee of the Faculty of Physical Therapy, Badr University in Cairo (Approval No. IRB00014233-54, date: 25 February 2025). Screening was performed at the outpatient pulmonology and rehabilitation clinics, and the eligibility of the potential participants was assessed by a combination of clinical and spirometry evaluation. Participants who fit the inclusion criteria were thoroughly informed of the objectives of the study, the procedures, and the consequences. Participants who were ready to join the study were given a written informed consent form, which they signed before joining the study. This research was conducted in accordance with the Declaration of Helsinki on ethical principles for medical research involving human subjects [22].

## 3. Design

The study design was a randomized, parallel-group, controlled trial aimed at comparing the impact of two therapeutic interventions on pulmonary function and chest expansion in patients with mild-to-moderate restrictive lung disease. Following baseline evaluation, eligible participants were assigned to Group A or Group B in a 1:1 ratio randomly after a concealed allocation procedure to minimize selection bias. The intervention was conducted over a period of eight weeks with a four-week follow-up to determine the sustainability of the effects of treatments. Outcome measures were FVC, FEV_1_, TLC, the FEV_1_/FVC ratio, and chest expansion at various thoracic levels at baseline, at 8 weeks (at the end of the interventions), and at the 4-week follow-up.

## 4. Subjects

A chest physician at the outpatient clinics screened the eligible participants. A total of 70 individuals were assessed for eligibility. Of these, 22 were excluded before randomization because they did not meet the eligibility criteria or declined participation. The remaining 48 eligible participants were randomized equally to Group A (n = 24) and Group B (n = 24). Participants were initially screened on the basis of reduced FVC and clinical assessment. A reduced FVC with a preserved or increased FEV_1_/FVC ratio was considered suggestive of a restrictive spirometric pattern but was not regarded as sufficient to confirm restriction. TLC was therefore assessed using whole-body plethysmography (MasterScreen Body system (M13.1; Jaeger/CareFusion Germany 234 GmbH, Höchberg, Germany). At baseline, 44 of the 48 randomized participants (91.7%) had TLC values < 80% of the predicted value, including 22/24 (91.7%) in Group A and 22/24 (91.7%) in Group B. TLC was recorded as a percentage of the predicted value. The same measurement method and equipment were used at baseline, immediately after the 8-week intervention, and at the 4-week follow-up to maintain consistency across assessment time points.

The inclusion criteria were that the participants were aged between 50 and 70 years and had a body mass index (BMI) between 18 and 30 kg/m^2^, in line with the suggestions of reducing the confounding effect of underweight and obesity on pulmonary functioning by the World Health Organization [23]. Participants had to be clinically stable, and they should not have had acute respiratory exacerbation in the past 4–6 weeks, as suggested in clinical trials of chronic respiratory illnesses and the Global Initiative of Chronic Obstructive Lung Disease [24]. The exclusion criteria were a history of recent acute cardiac events in the last six weeks, in accordance with common clinical trial safety guidelines of the International Council on Harmonizations of Technical Requirements on Pharmaceuticals to Human Use [25], unstable or decompensated congestive heart failure, active hemoptysis, malignancy, severe osteoporosis, or any other medical condition that may interfere with participation or jeopardize patient safety. Also, patients with severe gastroesophageal reflux disease or hiatus hernia were not included as they might have had contraindications to the therapeutic interventions applied.

**Figure 1:**
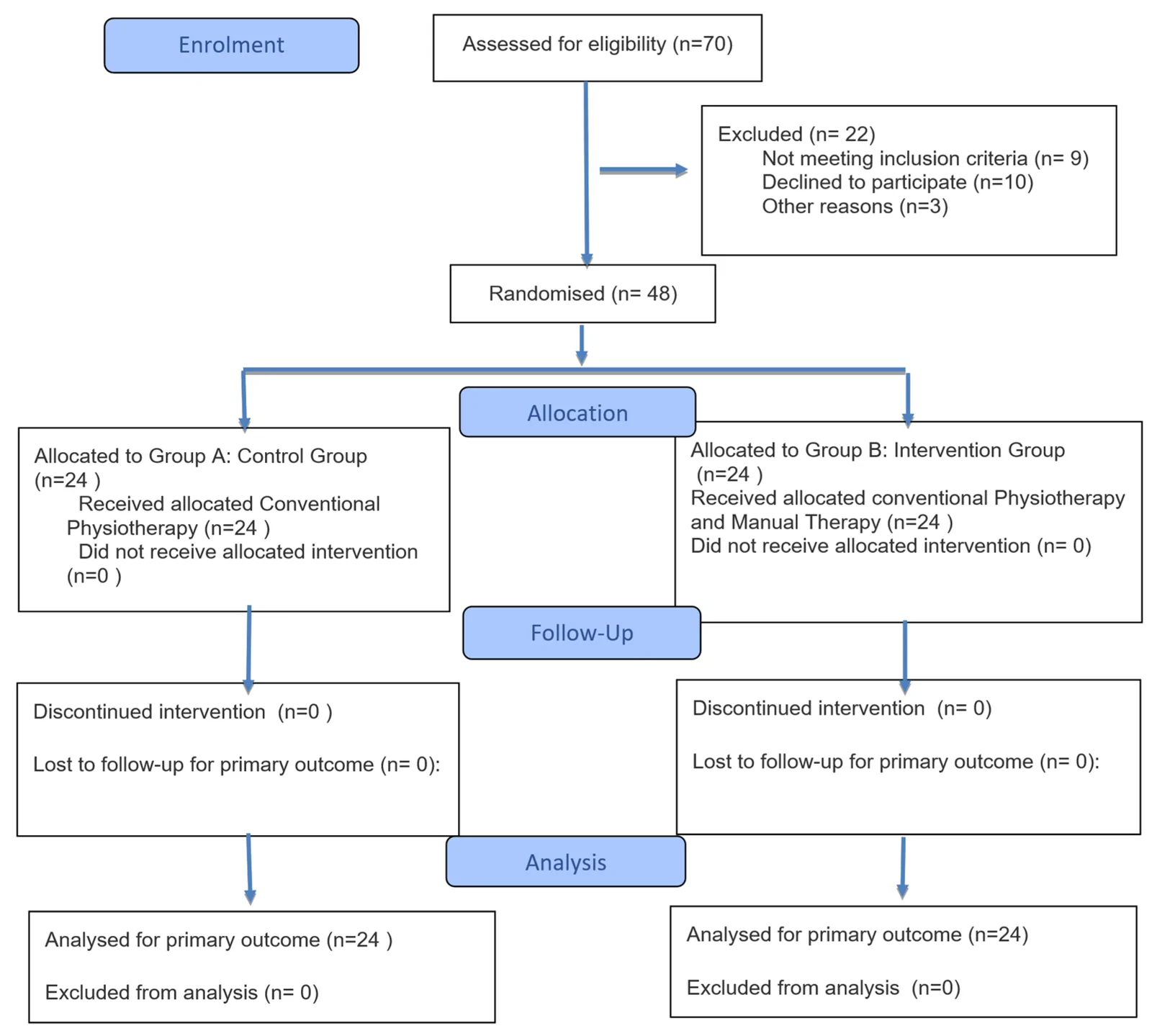
CONSORT Flow Diagram of Participants.

Following the use of the eligibility criteria, 48 of the participants were considered to fit the inclusion criteria and informed written consent was given before enrolling. Participants were then randomly assigned in a 1:1 proportion to two groups, Group A (n = 24) and Group B (n = 24), with computer-generated randomization sequence and allocation concealment ensured by sealed and opaque envelopes according to the recommendations of the CONSORT guidelines (CONSORT Group) (Figure 1) [26].

## 5. Sample Size Calculation

A repeated-measures analysis of variance (ANOVA) with a within-between interaction (group × time) was estimated using G*Power (version 3.1.9.7 Heinrich-Heine-Universität Düsseldorf, Düsseldorf, Germany). The two independent groups and three measurement time points (baseline, post-intervention at 8 weeks, and 4-week follow-up) were used to calculate it. The level of significance was set at 0.05 and the desired level of statistical power was 80%.

The group-time interaction was assumed to have a medium effect size (f = 0.25), as per usual standards and past clinical research based on pulmonary rehabilitation interventions [27]. A repeated measure correlation was conservatively set at *r* = 0.50, and the factor of non-sphericity was fixed at ε = 0.75 to address the possibility of violation of the assumption of sphericity.

According to these parameters, the minimum sample size was determined at 44 participants (22 per group) to identify statistically significant interaction. The sample size was expanded to 48 participants to compensate for a possible dropout rate of about 9%. Based on this, 48 individuals were selected and assigned to two groups (n = 24 for each group) randomly.

## 6. Randomization

After completion of the baseline assessments, eligible participants that met the inclusion criteria and had signed written informed consent were randomly assigned to two parallel groups. To maintain balance in the group sizes during the recruitment process, a block randomization system was used with variable block sizes (4 and 6) to reduce predictability of the allocation. An independent researcher who was not engaged in the participant recruitment, assessment, or intervention delivery generated the random sequence.

Consecutively numbered, sealed, opaque, and tamper-proof envelopes (SNOSE method) were used to achieve allocation concealment. The group assignment was kept in each envelope and opened in a sequence only after a baseline assessment was completed and the participant was found eligible. A researcher unrelated to the study team prepared and handled the envelopes to avoid any possible selection bias. Out of the 70 pre-screened participants, 22 were eliminated before randomization because they failed to meet the eligibility criteria or refused to participate. The rest of the eligible individuals (n = 48) were allocated randomly to two groups: Group A (n = 24) and Group B (n = 24).

Because of the nature of the interventions, participants and treating physiotherapists were not blinded to treatment allocation. However, the outcome assessor who performed the pulmonary-function and chest-expansion assessments was blinded to group allocation. The statistician/data analyst was also blinded to group identity during the primary analysis.

## 7. Primary Outcome Measures

The prespecified primary outcome was change in pulmonary function, operationalized as FVC (% predicted) at baseline, 8 weeks, and the 4-week follow-up. Secondary pulmonary outcomes included FEV_1_, FEV_1_/FVC, and chest expansion. These parameters were chosen as objective and clinically relevant measures of ventilatory functioning and as the basis of categorization of restrictive and obstructive pulmonary patterns based on the accepted standards [28].

1. A calibrated portable spirometer (Nisomed Contec automated spirometer 97 × 89 × 36 mm by Contec Medical Systems Co., Ltd., Qinhuangdao, China) was used to test the pulmonary function. Spirometry was performed according to the standardized recommendations of the American Thoracic Society and European Respiratory Society, which guarantee high validity and reproducibility of measurements. The spirometer had also shown good test–retest reliability, with intraclass correlation coefficients (ICCs) for key parameters ranging from 0.87 to 0.95 [29].

Participants were asked to sit upright with a nose clip on, inhale to full capacity, and then empty their lungs forcefully into the spirometer mouthpiece. Spirometry was performed according to standardized ATS/ERS procedures. At least three acceptable maneuvers were obtained, with additional attempts performed when required to satisfy repeatability criteria and subject tolerance. FVC and FEV_1_ were recorded according to the prespecified spirometry quality-control procedure, which is suggested by ATS/ERS standards to increase the reliability of measurements [30]. All the measurements were recorded at baseline, at 8 weeks after completion of the intervention, and at 4 weeks later as a follow-up.

2. Total lung capacity (TLC) was assessed using whole-body plethysmography (MasterScreen Body, Jaeger, Germany), a validated method for measuring static lung volumes based on changes in cabin pressure and thoracic gas volume. The equipment was calibrated according to standard procedures, and measurements followed ATS/ERS recommendations for acceptability and repeatability [31,32]. The same equipment and standardized procedures were used at baseline, after the 8-week intervention, and at the 4-week follow-up to ensure measurement consistency.

## 8. Secondary Outcome Measure

Chest expansion was the secondary outcome, which is evaluated as a measure of thoracic mobility and respiratory mechanics. Chest expansion was assessed with a non-stretchable measuring tape at three levels of the anatomy, including the 2nd intercostal space (upper chest), the 4th intercostal space (mid-chest), and the xiphoid process (lower chest) [33].

The measurements were performed at baseline, the 8-week intervention, and the 4-week follow-up. Measurements of maximal inspiration and maximum expiration were taken at every assessment time point, and the difference between these values was used to calculate chest expansion. The technique is common in clinical and research environments, and has shown reasonably good inter- and intrarater reliability under standardized procedures of Physical Therapy Assessment [34]. To reduce detection bias, all measurements were performed by a trained and blinded assessor. The levels of chest expansion were measured relative to the clinical norms, with normal chest expansion being 5–12.5 cm, and less or asymmetric expansion being a possible indication of impaired thoracic motion.

## 9. Interventions

Each intervention was directed in a 1:1 setting by a physiotherapist who had over 10 years of cardiopulmonary rehabilitation experience and was not aware of which group to include. The sessions were carried out in the morning before meals or at least 1.5 h after meals; both groups participated in supervised treatment, with three sessions per week for 8 consecutive weeks. In Group A, the conventional pulmonary rehabilitation program consisted of diaphragmatic breathing for approximately 5 min, segmental breathing for 5–7 min, active cycle of breathing technique (ACBT) for approximately 5 min, and volume-oriented incentive spirometry for 5–10 min, resulting in an overall conventional rehabilitation duration of approximately 20–27 min per session. In Group B, thoracic manual therapy was administered before the conventional pulmonary rehabilitation program and consisted of thoracic mobilization for approximately 5 min followed by myofascial release for approximately 9 min. Participants then completed the same conventional pulmonary rehabilitation program as Group A. Accordingly, the total supervised treatment duration was approximately 20–27 min per session for Group A and 34–41 min per session for Group B. The same treatment sequence and frequency were maintained throughout the intervention period, with minor adjustments within the prespecified time ranges according to participant tolerance.

**Group A (Control): Conventional Physiotherapy**

*Active control Group*

Participants in the active control group received a structured pulmonary rehabilitation program, including diaphragmatic breathing, segmental breathing exercises, active cycle of breathing techniques, and volume-oriented incentive spirometry. All the interventions were administered under the guidance of a trained respiratory physiotherapist with 10 years of experience to achieve standardization and protocol compliance across all participants.

Diaphragmatic Breathing

Diaphragmatic breathing was performed in a supported sitting position. Participants inhaled slowly through the nose for approximately 2–3 s and exhaled through pursed lips for approximately 4–6 s, while allowing passive abdominal movement. No external resistance was applied. The exercise was performed for 5 min per set for three sets.

Segmental Breathing

Segmental breathing was directed sequentially toward the apical, upper-lateral, lower-lateral, and posterior-basal thoracic regions. Three respiratory repetitions were performed at each target region, with gentle manual facilitation during inspiration when required. The total duration was approximately 5–7 min.

Active Cycle of Breathing Techniques (ACBT)

ACBT was conducted based on the typical elements, such as breathing regulation, thoracic expansion practice, and forced expiratory (huffing) techniques. The cycles were composed of relaxed breathing control, 3 deep thoracic expansion breaths and inspiratory hold of 2–3 s, followed by 1–2 maneuvers of huffing to aid the clearance of the airways. The session lasted about 5–7 min and was repeated 3 times.

Volume-oriented incentive spirometry

Volume-oriented incentive spirometry was performed by slow, sustained inspiration toward the prescribed visual target, followed by a comfortable breath hold and slow expiration. Each session included 10–15 repetitions.

The exercises were carried out in a specific order to maximize the effectiveness of respiration: diaphragmatic breathing, segmental breathing, ACBT, and volume-oriented incentive spirometry. A trained respiratory physiotherapist carried out all the interventions in order to ensure the proper execution of the intervention and reduce the variability among participants. The participants adhered to the protocol to improve reproducibility and minimize bias.

**Group B: Conventional Physiotherapy and Manual Therapy**

The experimental group underwent the same conventional pulmonary rehabilitation program as the control group, with a structured manual therapy program of thoracic mobilization and myofascial release techniques. All the interventions were conducted by a skilled physiotherapist who had 10 years of experience in musculoskeletal and cardiopulmonary rehabilitation.

*Thoracic Mobilization*

Thoracic joint mobilization was performed using the Maitland concept with Grade III oscillatory (large-amplitude movements that are applied against resistance) to enhance thoracic mobility and chest wall compliance [35,36]. Mobilization was applied in sitting and prone positions based on the comfort of participants and accessibility of therapists. Mobilizations were performed on the thoracic spinous processes (T2–T10) as central anterior–posterior (PA) mobilizations, and at the costovertebral joints to achieve segmental mobility using unilateral PA mobilizations. The oscillations were provided at a frequency of about 2–3 oscillations in a second and 30 s in one set and 3 sets in a segment were used on the targeted segment.

*Myofascial Release*

Myofascial release (MFR) procedures were directed to the important respiratory muscles and their related fascial arrangement, such as the diaphragm, pectoralis major and minor, sternocleidomastoid, scalene muscles, and cervical fascia. Each targeted muscle region was treated for approximately 3 min, according to participant tolerance. Constant hand pressure and mild stretching maneuvers were performed in the direction of tissue restriction until a release was felt [37].

The muscles of each group were treated for about 3 min, and the total duration of MFR was determined based on patient tolerance. Further chest wall mobilization was carried out laterally, along with a slow upper limb elevation to ease the fascial extension and enhance thoracic expansion.

The interventions were administered in a standard order to maximize the effect of treatment: thoracic mobilization, followed by myofascial release, and then the conventional exercises of the pulmonary rehabilitation program. The duration of each treatment session was about 30 min. Each session was performed directly under supervision to maintain appropriate technique and consistency across all participants. The treatment protocol was standardized in terms of technique, duration, and sequence but with slight changes in case of individual patient tolerance and clinical presentation.

## 10. Statistical Analysis

Statistical analyses were performed using IBM SPSS Statistics for Windows, version 26.0 (IBM Corp., Armonk, NY, USA). The estimation of the sample size was performed a priori by G*Power (version 3.1.9.7). The level of statistical significance was *p* < 0.05.

Continuous variables were reported as mean and standard deviation (SD) when the variables were normally distributed and median and interquartile range (IQR) when they were not normally distributed, and categorical variables were reported as frequencies and percentages. The Shapiro–Wilk test was used to test data normality and the Levene test was used to test homogeneity of variances. The Mauchly test was used to test the assumption of sphericity of repeated measures, and the Greenhouse–Geisser correction was used where the assumption was not met. Independent-samples *t*-tests were used to compare baseline demographic and clinical characteristics using continuous variables and chi-square test to compare categorical variables. The Mann–Whitney *U* test was an alternative nonparametric test employed in variables that did not satisfy the normality assumption.

The parameters of pulmonary functions (TLC, FEV_1_, FVC, FEV_1_/FVC) and chest expansion were compared with a mixed-design (two-way) repeated-measures analysis of variance (ANOVA), where one of the between-subject variables (groups: A vs. B) and one of the within-subject variables (time: at baseline, at 8 weeks of intervention, and at 4 weeks of follow-up) were used. Post hoc pair-wise comparisons with Bonferroni adjustment were conducted when there was any significant interaction or main effect. ANOVA effects were also reported as partial eta squared (η^2^p) and Cohen *d* was computed when necessary to compare two groups.

All analyses were performed using an intention-to-treat (ITT) approach. Missing data were addressed through multiple imputations with fully conditional specification, whereby five imputed datasets were created according to the available demographic and outcome variables.

## 11. Results

Seventy people were assessed for eligibility. Among them, 22 were excluded before randomization: nine for not meeting the inclusion criteria; 10 for declining to participate; and three for other reasons. Of the 48 eligible participants, the remaining 48 were randomized into two groups: Group A (n = 24) and Group B (n = 24) (Figure 1). Every participant underwent the interventions and was incorporated into the final analysis. No dropouts or missing data were recorded in the study period or in the follow-up. The trial was reported in accordance with the Consolidated Standards of Reporting Trials (CONSORT) statement for randomized controlled trials. The underlying clinical diagnoses responsible for the restrictive ventilatory impairment were recorded at baseline and were comparable between the two treatment groups. In both Group A and Group B, interstitial lung disease/pulmonary fibrosis was the most frequent underlying etiology (eight participants [33.3%] in each group), followed by chest-wall disorders (five [20.8%] in each group), pleural disease (four [16.7%] in each group), neuromuscular respiratory impairment (three [12.5%] in each group), post-tuberculosis/post-infectious restrictive changes (two [8.3%] in each group), and other chronic restrictive conditions (two [8.3%] in each group). Overall, these diagnoses accounted for 16 (33.3%), 10 (20.8%), eight (16.7%), six (12.5%), four (8.3%), and four (8.3%) of the 48 participants, respectively. The sex distribution was comparable between the two treatment groups. Group A included 13 male participants (54.2%) and 11 female participants (45.8%), whereas Group B included 12 male participants (50.0%) and 12 female participants (50.0%). No statistically significant difference was observed in sex distribution between the groups (χ^2^ = 0.083, df = 1, *p* = 0.773). Table 1 summarizes the baseline characteristics of the sample.

Group A showed significant baseline-to-8-week improvements in FVC, FEV_1_, and TLC, with *p*-values ranging from 0.001 to 0.003. These changes were not statistically significant between 8 weeks and follow-up. Overall, FVC, FEV_1_, and TLC remained significantly improved from baseline to follow-up, with small-to-moderate effect sizes (FVC: MD = 5.9, 95% CI: 3.6–8.2, d = 0.71, *p* = 0.001; FEV_1_: MD = 4.5, 95% CI: 2.4–6.6, d = 0.60, *p* = 0.001; TLC: MD = 4.3, 95% CI: 2.4–6.2, d = 0.55, *p* = 0.002). Greater improvements were observed in Group B. Baseline-to-8-week changes were significant for FVC (MD = 13.4, 95% CI: 10.2–16.6, d = 1.74, *p* < 0.001), FEV_1_ (MD = 10.8, 95% CI: 7.6–14.0, d = 1.43, *p* < 0.001), and TLC (MD = 12.0, 95% CI: 8.9–15.1, d = 1.50, *p* < 0.001). The changes from 8 weeks to follow-up were small but significant for FVC (*p* = 0.030), FEV_1_ (*p* = 0.025), and TLC (*p* = 0.045). The overall baseline-to-follow-up improvements were large, with FVC increasing by 14.8 (95% CI: 11.6–18.0, d = 1.91, *p* < 0.001), FEV_1_ by 12.2 (95% CI: 9.0–15.4, d = 1.62, *p* < 0.001), and TLC by 13.2 (95% CI: 10.1–16.3, d = 1.65, *p* < 0.001) (Table 2).

Within-group changes in the FEV_1_/FVC ratio were significant in both groups, although the magnitude of change was greater in Group B. In Group A, the baseline-to-8-week change was small but significant (MD = 0.01, 95% CI: 0.002–0.018, d = 0.33, *p* = 0.010), with no significant change between 8 weeks and follow-up (*p* = 0.900). The baseline-to-follow-up improvement remained significant (MD = 0.01, 95% CI: 0.002–0.018, d = 0.34, *p* = 0.008). In Group B, the baseline-to-8-week change was greater (MD = 0.04, 95% CI: 0.028–0.052, d = 1.33, *p* < 0.001), followed by a small but significant change between 8 weeks and follow-up (MD = 0.01, 95% CI: 0.004–0.016, d = 0.33, *p* = 0.010). Overall, the baseline-to-follow-up change in Group B was significant and large (MD = 0.05, 95% CI: 0.036–0.064, d = 1.67, *p* < 0.001) (Table 2).

Both groups showed significant changes in chest expansion, with larger effect sizes in Group B. In Group A, chest expansion at the second ICS improved from baseline to 8 weeks (MD = 0.37 cm, 95% CI: 0.20–0.54, d = 0.90, *p* = 0.001) and remained significantly improved at follow-up (MD = 0.39 cm, 95% CI: 0.22–0.56, d = 0.95, *p* = 0.001). Similar significant baseline-to-follow-up improvements were observed at the fourth ICS (MD = 0.45 cm, 95% CI: 0.23–0.67, d = 0.99, *p* = 0.001) and xiphoid level (MD = 0.47 cm, 95% CI: 0.25–0.69, d = 0.90, *p* = 0.001). Changes between 8 weeks and follow-up were not statistically significant at these measurement levels (Table 2).

Chest expansion showed greater improvements in Group B. At the second ICS, the change from baseline to 8 weeks was 0.77 cm (95% CI: 0.56–0.98, d = 1.54, *p* < 0.001), with an overall baseline-to-follow-up improvement of 0.84 cm (95% CI: 0.63–1.05, d = 1.68, *p* < 0.001). Similar significant improvements were found at the fourth ICS, with a baseline-to-8-week change of 0.97 cm (95% CI: 0.72–1.22, d = 1.84, *p* < 0.001) and a baseline-to-follow-up change of 1.06 cm (95% CI: 0.81–1.31, d = 2.00, *p* < 0.001). At the xiphoid level, the change from baseline to 8 weeks was 1.18 cm (95% CI: 0.93–1.43, d = 2.08, *p* < 0.001), increasing to 1.28 cm at follow-up (95% CI: 1.03–1.53, d = 2.30, *p* < 0.001). The changes from 8 weeks to follow-up were small, with significant changes at the fourth ICS (*p* = 0.025) and xiphoid level (*p* = 0.020), but not at the second ICS (*p* = 0.080) (Table 2).

Both groups showed significant improvements in pulmonary function and chest expansion during the intervention period. Group B demonstrated greater changes and larger effect sizes than Group A, particularly for FVC, FEV_1_, TLC, and chest expansion, indicating a greater improvement following the addition of thoracic mobilization and myofascial release to conventional pulmonary rehabilitation. These improvements were maintained at follow-up, with only small additional changes observed between 8 weeks and follow-up (Table 2).

**Figure 2:**
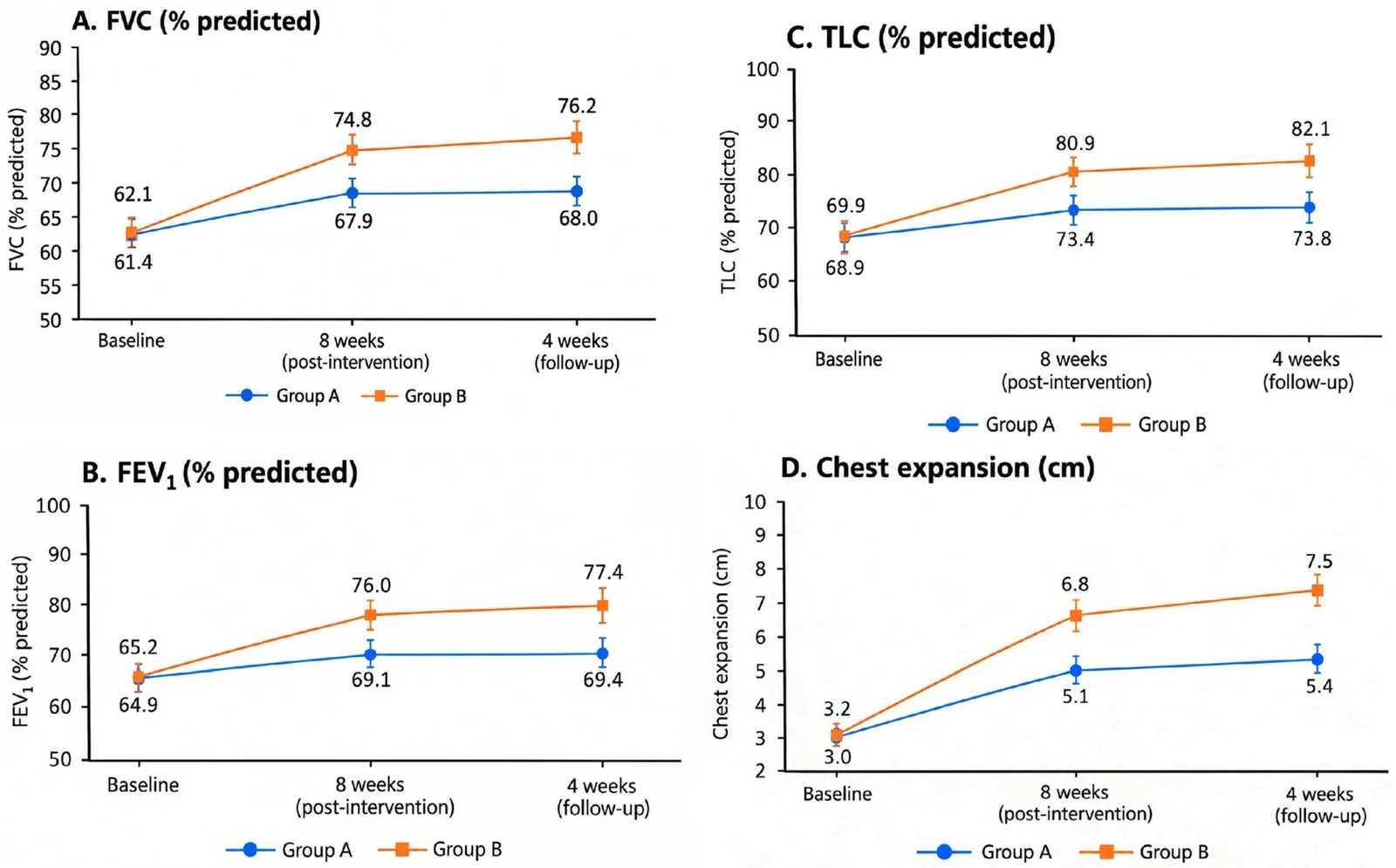
Mean (95% CI) changes in (**A**) FVC (% predicted), (**B**) FEV_1_ (% predicted), (**C**) TLC (% predicted), (**D**) Chest expansion (cm) at baseline, 8-week post-intervention, and 4-week follow-up in Groups A and B.

Between-group comparisons demonstrated significant differences in favor of Group B at 8 weeks and at follow-up for all pulmonary function and chest expansion outcomes (Table 3). At 8 weeks, Group B showed significantly higher FVC than Group A (74.8 ± 7.1% vs. 67.9 ± 7.5% predicted; MD = 6.90, 95% CI: 3.44–10.36; *p* = 0.002; Cohen’s *d* = 0.92). Similarly, FEV_1_ was significantly higher in Group B (76.0 ± 7.5% vs. 69.1 ± 6.9% predicted; MD = 6.90, 95% CI: 2.93–10.87; *p* = 0.002; Cohen’s *d* = 0.96), as was TLC (80.9 ± 7.7% vs. 73.4 ± 7.3% predicted; MD = 7.50, 95% CI: 3.42–11.58; *p* = 0.001; Cohen’s *d* = 1.00). These between-group differences were maintained or increased at the 4-week follow-up, with higher FVC (76.2 ± 6.8% vs. 68.0 ± 7.4% predicted; MD = 8.20, 95% CI: 4.65–11.74; *p* < 0.001; Cohen’s *d* = 1.09), FEV_1_ (77.4 ± 7.0% vs. 69.4 ± 6.7% predicted; MD = 8.00, 95% CI: 4.28–11.73; *p* < 0.001; Cohen’s *d* = 1.17), and TLC (82.1 ± 7.2% vs. 73.8 ± 7.0% predicted; MD = 8.30, 95% CI: 4.44–12.16; *p* < 0.001; Cohen’s *d* = 1.17) in Group B compared with Group A (Table 3 and Figure 2).

Significant between-group differences were also observed in chest expansion at all three measurement levels. At 8 weeks, Group B demonstrated greater chest expansion at the second intercostal space (2.25 ± 0.44 vs. 1.82 ± 0.41 cm; MD = 0.43, 95% CI: 0.199–0.661; *p* = 0.006; Cohen’s *d* = 1.01), the fourth intercostal space (2.76 ± 0.52 vs. 2.18 ± 0.47 cm; MD = 0.58, 95% CI: 0.311–0.849; *p* = 0.002; Cohen’s *d* = 1.17), and the xiphoid level (3.18 ± 0.58 vs. 2.48 ± 0.56 cm; MD = 0.70, 95% CI: 0.390–1.010; *p* = 0.001; Cohen’s *d* = 1.23). These differences were sustained at follow-up, with greater expansion in Group B at the second intercostal space (2.32 ± 0.46 vs. 1.84 ± 0.42 cm; MD = 0.48, 95% CI: 0.241–0.719; *p* = 0.003; Cohen’s *d* = 1.09), fourth intercostal space (2.85 ± 0.51 vs. 2.21 ± 0.46 cm; MD = 0.64, 95% CI: 0.376–0.904; *p* < 0.001; Cohen’s *d* = 1.32), and xiphoid level (3.28 ± 0.57 vs. 2.52 ± 0.55 cm; MD = 0.76, 95% CI: 0.456–1.064; *p* < 0.001; Cohen’s *d* = 1.36). Overall, the findings indicate that the addition of thoracic mobilization and myofascial release to conventional pulmonary rehabilitation was associated with greater improvements in pulmonary function and thoracic expansion than conventional pulmonary rehabilitation alone, with between-group differences evident at 8 weeks and maintained at the 4-week follow-up (Table 3 and Figure 2).

The prespecified primary treatment effect was evaluated using the group × time interaction, which assessed whether the trajectory of FVC (% predicted) differed between the two treatment groups across baseline, post-intervention, and follow-up assessments. Significant group × time interactions were observed for FVC and all secondary pulmonary-function and chest-expansion outcomes (all *p* < 0.001), indicating that the magnitude and pattern of change over time differed between the two groups. The between-group comparisons at the post-intervention and follow-up assessments showed greater values in Group B than in Group A for the pulmonary-function and chest-expansion outcomes. These findings support a greater treatment-associated change in Group B compared with conventional pulmonary rehabilitation alone.

The principal treatment effect was assessed using the group × time interaction across baseline, post-intervention (8 weeks), and follow-up assessments. Significant group × time interactions were observed for FVC (F(2, 92) = 885.012, *p* < 0.001, partial η^2^ = 0.951), FEV_1_ (F(2, 92) = 983.222, *p* < 0.001, partial η^2^ = 0.955), TLC (F(2, 92) = 1587.526, *p* < 0.001, partial η^2^ = 0.972), FEV_1_/FVC (F(2, 92) = 106.028, *p* < 0.001, partial η^2^ = 0.697), chest expansion at the second intercostal space (F(2, 92) = 1068.723, *p* < 0.001, partial η^2^ = 0.959), fourth intercostal space (F(2, 92) = 865.291, *p* < 0.001, partial η^2^ = 0.950), and xiphoid level (F(2, 92) = 2444.152, *p* < 0.001, partial η^2^ = 0.982). These findings indicated that changes over time differed significantly between the two treatment groups, with greater improvements generally observed in Group B. FVC increased from 61.4% at baseline to 74.8% at 8 weeks and 76.2% at follow-up in Group B, compared with increases from 62.1% to 67.9% and 68.0%, respectively (Table 4).

Attendance was monitored throughout the intervention period. In Group A, 23 of the 24 participants (95.8%) completed the prescribed treatment sessions, with a mean adherence of 95.8 ± 6.2%. In Group B, 22 of 24 participants (91.7%) completed the prescribed sessions, with a mean adherence of 91.7 ± 9.1%. One protocol deviation occurred in each group. No participants required additional respiratory interventions during the study period. Home-exercise participation was reported by 20 participants (83.3%) in Group A and 19 participants (79.2%) in Group B. One adverse event was reported in each group (4.2%); neither event resulted in treatment discontinuation. Overall, treatment adherence was high, and no major safety concerns were observed.

## 12. Discussion

The primary finding of this randomized controlled trial was that adding thoracic manual therapy to conventional pulmonary rehabilitation was associated with a greater improvement in FVC (% predicted) over time than conventional pulmonary rehabilitation alone. Significant group × time interactions were also observed for FEV_1_, TLC, FEV_1_/FVC, and chest expansion at the second and fourth intercostal spaces and xiphoid level. The direction of these effects was consistent with greater improvement in Group B, and the between-group differences observed after the 8-week intervention were maintained at the 4-week follow-up. These findings suggest that the addition of thoracic manual therapy may provide an additional benefit for selected pulmonary-function and thoracic-mobility outcomes in adults with restrictive ventilatory impairment.

These greater changes in lung functioning are indicative of both groups having a greater ventilatory capacity, which can probably be attributed to increased lung volumes, better alveolar ventilation, and greater respiratory musculature [38]. The greater improvements in Group B may be due to theory-based biomechanical or neurophysiological changes, such as increased thoracic mobility and changes in the mechanics of the chest wall following manual mobilization and myofascial release [39,40]. Other factors, including decreased mechanical resistance to ventilation, may be responsible for these; however, these were not measured directly in our trial, which did not measure respiratory muscle strength, diaphragmatic excursion, thoracic compliance, alveolar recruitment, airway patency or ventilation–perfusion matching. Hence these suggested physiological mechanisms should be considered as speculative or as possible mechanisms and not confirmed mechanisms, which indirectly facilitates changes in FEV_1_ and the FEV_1_/FVC ratio. Taken together, these mechanisms probably underlie the greater and more prolonged improvements in pulmonary function and chest expansion in Group B. Both the groups experienced significant improvements in chest expansion, but this represents better rib cage mobility and intercostal muscle work. The increased chest expansion correlates with the increased diaphragmatic excursion and work of breathing, as well as the improved ventilation–perfusion matching, all of which favor functional respiratory efficiency [41].

The results of our study were consistent with previous studies on the advantages of organized respiratory and thoracic exercises. The respiratory exercises were found to be effective in enhancing FVC and FEV_1_ in restrictive ventilatory disorders patients (*p* < 0.01) [42]. Thoracic mobility exercises enhanced chest expansion and functional capacity in adults with diminished thoracic compliance [43]. In the same way, multi-component respiratory training resulted in greater improvements in pulmonary function than standard interventions [44,45]. The high effect sizes in Group B of this study indicate that the strength, specificity and comprehensiveness of the intervention have a strong impact.

The study results indicated that multi-component interventions have a significant potential to enhance pulmonary functioning and thoracic mobility. Such interventions should be considered by clinicians to treat patients with restrictive ventilatory patterns, who have limited lung volumes or conditions that prevent thoracic expansion. Functional capacity can be improved with better pulmonary performance and greater chest expansion techniques to decrease exertional dyspnea and improve quality of life, especially in elderly individuals or those with long-term respiratory impairments [46]. The findings at the 4-week follow-up indicate short-term maintenance of the post-intervention improvements in pulmonary function and chest expansion, though longer follow-up periods are required to evaluate whether these effects persist over the long term. The additional benefit seen in the experimental group could be due to the passive soft-tissue resistance reduction and acute increase in thoracic flexibility [47]. No such explanations could be made, however, as the localized circulation, lymphatic return, delivery of oxygen and the forces of respiratory muscles were not assessed [48]; this is only a hypothesis and needs to be verified in further trials using physiological instrumentation [49]. Providing MRT on the basis of soft tissue alone does not enhance exercise performance, dyspnea levels, and lung functions. Nonetheless, when combined with a joint-based MRT, dyspnea levels and breathing muscle stretching with increased thoracic cage flexibility are observed [50]. The observed improvements may be related to enhanced thoracic mobility, altered chest-wall mechanics, improved breathing-pattern coordination, and reduced soft-tissue restriction. However, respiratory muscle strength, diaphragmatic excursion, alveolar recruitment, airway patency, and ventilation–perfusion matching were not directly measured in this study and therefore remain possible rather than demonstrated mechanisms [51].

The greater improvement observed in Group B may be related to changes in thoracic mobility and chest-wall mechanics produced by the addition of thoracic mobilization and myofascial release. The increase in measured chest expansion provides objective evidence of improved thoracic excursion, although the underlying physiological mechanisms cannot be determined from the present measurements. In particular, respiratory muscle strength, diaphragmatic excursion, thoracic compliance, alveolar recruitment, airway patency, and ventilation–perfusion matching were not directly assessed. Therefore, these mechanisms should be regarded as possible explanations rather than demonstrated mediators of the observed changes. Future studies incorporating direct measurements of respiratory muscle strength, diaphragmatic movement, and thoracic mechanics are required to clarify the mechanisms underlying the observed treatment effects.

The strengths of this trial were its randomized design, sufficient sample size, objective outcome measures, and repeated assessments at baseline, post-intervention, and follow-up. The study provided solid evidence of the effectiveness of multi-component interventions in enhancing lung performance and chest volume. The limitations include the single-center-based research design, which might limit the generalizability. Although the study produced functional respiratory results, it failed to directly measure respiratory muscle strength, diaphragmatic excursion, or thoracic compliance, which might explain the mechanisms of observed improvements. These measurements should be included in future studies and long-term follow-up should be considered. The persistence of the observed effects during the four-week follow-up suggests short-term maintenance of treatment-related changes; longer follow-up is required to determine whether these effects persist over subsequent months. The restrictive ventilatory impairment represented a clinically heterogeneous population, and diagnosis-specific subgroup analyses were not designed to determine whether treatment effects differed according to etiology. Chest expansion was measured using a tape-based technique and may be subject to measurement variability, and multiple outcomes increase the possibility of type I error. The total supervised treatment time differed between groups. Group A received approximately 20–27 min of supervised treatment per session, whereas Group B received approximately 34–41 min because the manual therapy component was added to the same conventional pulmonary rehabilitation program. Consequently, the observed between-group differences cannot be attributed exclusively to the specific manual therapy techniques, because the experimental group also received a greater amount of therapist contact and treatment time. Future trials should consider time-matched control interventions to isolate the specific contribution of thoracic manual therapy. These limitations should be considered in future studies.

## 13. Conclusions

In adults with restrictive ventilatory impairment, adding thoracic manual therapy to conventional pulmonary rehabilitation was associated with greater improvements in selected pulmonary-function and chest-expansion measures than conventional pulmonary rehabilitation alone. These differences were maintained during the four-week follow-up period. Larger, adequately powered trials with diagnosis-specific populations, longer follow-up, and patient-centered outcomes are needed to confirm these findings and determine their clinical relevance.

## Figures and Tables

**Table 1:** Baseline Characteristics of Participants.

Variable	Group A (n = 24)	Group B (n = 24)	*p*-Value
Age (years)	60.85 ± 5.40	61.12 ± 4.98	0.78
BMI (kg/m^2^)	23.18 ± 1.85	23.42 ± 1.79	0.56
FVC (%)	62.1 ± 8.4	61.4 ± 7.9	0.73
FEV_1_ (%)	64.9 ± 7.5	65.2 ± 8.0	0.88
TLC (%)	69.5 ± 7.8	68.9 ± 8.1	0.81
Chest expansion (2nd ICS) (cm)	1.48 ± 0.41	1.45 ± 0.39	0.75
Chest expansion (4th ICS) (cm)	1.76 ± 0.46	1.72 ± 0.44	0.71
Chest expansion (xiphoid) (cm)	2.05 ± 0.54	2.00 ± 0.50	0.68

Values are shown as mean ± standard deviation; BMI; Body Mass Index; FVC; Forced Vital Capacity; FEV_1_ = Forced Expiratory Volume in one second; TLC; Total Lung Capacity. *p*-value; Probability Value.

**Table 2:** Within-group changes from baseline to 8 weeks and follow-up.

Outcome	Group	Time Comparison	MD	95% CI	Cohen’s d	*p*-Value
FVC (% predicted)	Group A	Baseline vs. 8 weeks	5.8	3.5 to 8.1	0.69	0.001
		8 weeks vs. follow-up	0.1	−0.8 to 1.0	0.02	0.850
		Baseline vs. follow-up	5.9	3.6 to 8.2	0.71	0.001
	Group B	Baseline vs. 8 weeks	13.4	10.2 to 16.6	1.74	<0.001
		8 weeks vs. follow-up	1.4	0.2 to 2.6	0.18	0.030
		Baseline vs. follow-up	14.8	11.6 to 18.0	1.91	<0.001
FEV_1_ (% predicted)	Group A	Baseline vs. 8 weeks	4.2	2.1 to 6.3	0.56	0.002
		8 weeks vs. follow-up	0.3	−0.5 to 1.1	0.04	0.450
		Baseline vs. follow-up	4.5	2.4 to 6.6	0.60	0.001
	Group B	Baseline vs. 8 weeks	10.8	7.6 to 14.0	1.43	<0.001
		8 weeks vs. follow-up	1.4	0.2 to 2.6	0.19	0.025
		Baseline vs. follow-up	12.2	9.0 to 15.4	1.62	<0.001
TLC (% predicted)	Group A	Baseline vs. 8 weeks	3.9	2.0 to 5.8	0.50	0.003
		8 weeks vs. follow-up	0.4	−0.5 to 1.3	0.05	0.400
		Baseline vs. follow-up	4.3	2.4 to 6.2	0.55	0.002
	Group B	Baseline vs. 8 weeks	12.0	8.9 to 15.1	1.50	<0.001
		8 weeks vs. follow-up	1.2	0.0 to 2.4	0.15	0.045
		Baseline vs. follow-up	13.2	10.1 to 16.3	1.65	<0.001
FEV_1_/FVC (ratio)	Group A	Baseline vs. 8 weeks	0.01	0.002 to 0.018	0.33	0.010
		8 weeks vs. follow-up	0.00	−0.001 to 0.001	0.00	0.900
		Baseline vs. follow-up	0.01	0.002 to 0.018	0.34	0.008
	Group B	Baseline vs. 8 weeks	0.04	0.028 to 0.052	1.33	<0.001
		8 weeks vs. follow-up	0.01	0.004 to 0.016	0.33	0.010
		Baseline vs. follow-up	0.05	0.036 to 0.064	1.67	<0.001
Chest expansion—2nd ICS (cm)	Group A	Baseline vs. 8 weeks	0.37	0.20 to 0.54	0.90	0.001
		8 weeks vs. follow-up	0.02	−0.06 to 0.10	0.05	0.400
		Baseline vs. follow-up	0.39	0.22 to 0.56	0.95	0.001
	Group B	Baseline vs. 8 weeks	0.77	0.56 to 0.98	1.54	<0.001
		8 weeks vs. follow-up	0.07	−0.01 to 0.15	0.13	0.080
		Baseline vs. follow-up	0.84	0.63 to 1.05	1.68	<0.001
Chest expansion—4th ICS (cm)	Group A	Baseline vs. 8 weeks	0.42	0.20 to 0.64	0.92	0.001
		8 weeks vs. follow-up	0.03	−0.05 to 0.11	0.07	0.350
		Baseline vs. follow-up	0.45	0.23 to 0.67	0.99	0.001
	Group B	Baseline vs. 8 weeks	0.97	0.72 to 1.22	1.84	<0.001
		8 weeks vs. follow-up	0.09	0.01 to 0.17	0.18	0.025
		Baseline vs. follow-up	1.06	0.81 to 1.31	2.00	<0.001
Chest expansion—xiphoid (cm)	Group A	Baseline vs. 8 weeks	0.43	0.21 to 0.65	0.82	0.001
		8 weeks vs. follow-up	0.04	−0.06 to 0.14	0.08	0.300
		Baseline vs. follow-up	0.47	0.25 to 0.69	0.90	0.001
	Group B	Baseline vs. 8 weeks	1.18	0.93 to 1.43	2.08	<0.001
		8 weeks vs. follow-up	0.10	0.02 to 0.18	0.20	0.020
		Baseline vs. follow-up	1.28	1.03 to 1.53	2.30	<0.001

MD; mean difference of within-group change between the time points; 95% CI; confidence interval of the difference in mean; Cohen’s *d*; standardized effect size of within-group change (0.2 small, 0.5 medium, 0.8 large); *p*-values are associated with pair-wise comparison within the group; FVC; Forced Vital Capacity; FEV_1_; Forced Expiratory Volume in 1 s; TLC; Total Lung Capacity; ICS; Intercostal Space; MD positive values indicated an improvement compared with the previous timepoint.

**Table 3:** Between-Group Comparison of Pulmonary Function and Chest Expansion Outcomes Across Study Timepoints.

Outcome	Time	Group A Mean ± SD	Group B Mean ± SD	MD (B − A)	95% CI	Cohen’s *d*	*p*-Value
FVC (% predicted)	Baseline	62.1 ± 8.4	61.4 ± 7.9	−0.70	−5.19 to 3.79	−0.09	0.768
	8 weeks	67.9 ± 7.5	74.8 ± 7.1	6.90	3.44 to 10.36	0.92	0.002
	Follow-up	68.0 ± 7.4	76.2 ± 6.8	8.20	4.65 to 11.74	1.09	<0.001
FEV_1_ (% predicted)	Baseline	64.9 ± 7.5	65.2 ± 8.0	0.30	−4.28 to 4.88	0.04	0.894
	8 weeks	69.1 ± 6.9	76.0 ± 7.5	6.90	2.93 to 10.87	0.96	0.002
	Follow-up	69.4 ± 6.7	77.4 ± 7.0	8.00	4.28 to 11.73	1.17	<0.001
TLC (% predicted)	Baseline	69.5 ± 7.8	68.9 ± 8.1	−0.60	−4.92 to 3.72	−0.08	0.796
	8 weeks	73.4 ± 7.3	80.9 ± 7.7	7.50	3.42 to 11.58	1.00	0.001
	Follow-up	73.8 ± 7.0	82.1 ± 7.2	8.30	4.44 to 12.16	1.17	<0.001
FEV_1_/FVC (ratio)	Baseline	0.78 ± 0.03	0.78 ± 0.04	0.00	−0.019 to 0.019	0.00	1.000
	8 weeks	0.79 ± 0.03	0.82 ± 0.03	0.03	0.014 to 0.046	1.00	0.001
	Follow-up	0.79 ± 0.03	0.83 ± 0.02	0.04	0.026 to 0.054	1.57	<0.001
Chest expansion—2nd ICS (cm)	Baseline	1.48 ± 0.41	1.45 ± 0.39	−0.03	−0.26 to 0.20	−0.07	0.80
	8 weeks	1.82 ± 0.41	2.25 ± 0.44	0.43	0.199 to 0.661	1.01	0.006
	Follow-up	1.84 ± 0.42	2.32 ± 0.46	0.48	0.241 to 0.719	1.09	0.003
Chest expansion—4th ICS (cm)	Baseline	1.76 ± 0.46	1.72 ± 0.44	−0.04	−0.30 to 0.22	−0.09	0.76
	8 weeks	2.18 ± 0.47	2.76 ± 0.52	0.58	0.311 to 0.849	1.17	0.002
	Follow-up	2.21 ± 0.46	2.85 ± 0.51	0.64	0.376 to 0.904	1.32	<0.001
Chest expansion—Xiphoid (cm)	Baseline	2.05 ± 0.54	2.00 ± 0.50	−0.05	−0.35 to 0.25	−0.10	0.73
	8 weeks	2.48 ± 0.56	3.18 ± 0.58	0.70	0.390 to 1.010	1.23	0.001
	Follow-up	2.52 ± 0.55	3.28 ± 0.57	0.76	0.456 to 1.064	1.36	<0.001

FVC; Forced Vital Capacity; FEV_1_ = Forced Expiratory Volume in 1 s; TLC = Total Lung Capacity; IC = Intercostal Space; SD = Standard Deviation, MD = mean difference between groups; 95% CI = 95% confidence interval of the mean difference; Cohen’s *d* = the standardized effect size (0.2, 0.5 and 0.8 are small, medium and large effects, respectively).

**Table 4:** Two-way repeated-measures ANOVA for pulmonary function and chest expansion outcomes.

Outcome	Effect	F (df_1_, df_2_)	*p*-Value	Partial η^2^ (ηp^2^)
FVC (% predicted)	Group	14.92 (1, 46)	0.001	0.245
	Time	43.01 (2, 92)	<0.001	0.483
	Group × Time	34.39 (2, 92)	<0.001	0.428
FEV_1_ (% predicted)	Group	12.84 (1, 46)	0.002	0.218
	Time	38.40 (2, 92)	<0.001	0.455
	Group × Time	32.92 (2, 92)	<0.001	0.417
TLC (% predicted)	Group	11.96 (1, 46)	0.003	0.206
	Time	41.96 (2, 92)	<0.001	0.477
	Group × Time	35.61 (2, 92)	<0.001	0.436
FEV_1_/FVC (ratio)	Group	7.88 (1, 46)	0.012	0.146
	Time	20.50 (2, 92)	<0.001	0.308
	Group × Time	25.04 (2, 92)	<0.001	0.352
Chest expansion—2nd ICS (cm)	Group	11.87 (1, 46)	0.004	0.205
	Time	46.98 (2, 92)	<0.001	0.505
	Group × Time	34.39 (2, 92)	<0.001	0.428
Chest expansion—4th ICS (cm)	Group	13.94 (1, 46)	0.002	0.233
	Time	48.96 (2, 92)	<0.001	0.516
	Group × Time	39.44 (2, 92)	<0.001	0.462
Chest expansion—Xiphoid (cm)	Group	14.92 (1, 46)	0.001	0.245
	Time	50.05 (2, 92)	<0.001	0.521
	Group × Time	41.10 (2, 92)	<0.001	0.472

FVC = Forced Vital Capacity; FEV_1_ = Forced Expiratory Volume in one second; TLC = Total Lung Capacity; ICS = Intercostal Space; df_1_ = degrees of freedom of the effect, df_2_ = degrees of freedom of the error term in the repeated-measures ANOVA; ηp^2^ = a partial eta squared, denoted as an effect size index (0.01 = small, 0.06 = medium, 0.14 = large). The group effect also quantifies inter-subject differences in treatment between the intervention and control conditions, yet the time effect quantifies within-subject differences at the three measurement points (baseline, post-treatment and follow-up). Group × Time interaction indicates the difference between the change over time in groups.

## Data Availability

The data are available at: https://doi.org/10.6084/m9.figshare.33278130.
